# Impact of a passive upper-body exoskeleton on muscular activity and precision in overhead single and dual tasks: an explorative randomized crossover study

**DOI:** 10.3389/fneur.2024.1405473

**Published:** 2024-06-28

**Authors:** Julia Gräf, Sidney Grospretre, Andreas Argubi-Wollesen, Bettina Wollesen

**Affiliations:** ^1^Human Movement Science, University of Hamburg, Hamburg, Germany; ^2^Laboratory Culture Sport Health and Society (C3S-UR 4660), University of Franche-Comté, Besançon, France; ^3^Institut Universitaire de France (IUF), Paris, France; ^4^exoIQ GmbH, Hamburg, Germany

**Keywords:** exoskeleton, muscle activity, Fitts’ task, dual task (DT), overhead work

## Abstract

**Introduction:**

Tasks performed at or above head height in industrial workplaces pose a significant challenge due to their association with musculoskeletal disorders. Upper-body exoskeletons have been identified as a potential solution for mitigating musculoskeletal loads and fighting against excessive muscular fatigue. However, the influence of such support on fine motor control, as well as on cognitive-motor interference, has received limited attention thus far. Therefore, this crossover randomized study aimed to investigate the impact of the use of a passive upper-body exoskeleton in the presence of muscular fatigue or not. Additionally, focusing on differences between single (ST) and dual (DT) industrial tasks consisting of overhead speed and accuracy exercises.

**Methods:**

In both scenarios, *N* = 10 participants (5 male/5 female) engaged in an overhead precision task using a nail gun to precisely target specific areas on three differently sized regions, based on Fitts’ law paradigm (speed-accuracy trade-off task). This was done with and without the passive upper-body exoskeleton, before and immediately after a fatiguing exercise of shoulder and leg muscles. In addition, a second task (dual-task, DT) was carried out in which the occurrence of an auditory signal had to be counted. The main outcomes were muscular activation of the shoulder girdle as well as the time to perform speed-accuracy tasks of different difficulty indexes (calculated by means of Fitts’ law).

**Results and discussion:**

In the absence of fatigue, the exoskeleton did not affect the speed-accuracy trade-off management of participants in the single task, but it did in the dual-task conditions. However, after muscle fatigue, the speed-accuracy trade-off was differently affected when comparing its execution with or without the exoskeleton. In general, the dual task resulted in longer times to perform the different tasks, whether it was with or without the exoskeleton. Furthermore, the use of the exoskeleton decreased muscle activity, which is associated with less physical effort, but only significantly for the *M. deltoideus* and *M. trapezius* when compared by tasks. Overall, these study findings highlight the potential supportive effects of using an upper-body exoskeleton for industrial overhead tasks.

## 1 Introduction

In industries such as manufacturing, construction, and logistics, workers often engage in repetitive and physically demanding tasks that can lead to musculoskeletal injuries. Work-related musculoskeletal disorders (WMSD) and related health consequences continue to be major problems in the construction and industrial sectors (1). They are the most common cause of work incapacity, limited abilities, and premature disability. WMSDs are the leading cause of around 17.5% (Germany) of all days of absenteeism (2). WMSDs are caused by physical stress, e.g., lifting and carrying heavy loads and sustained strenuous postures (3). Especially overhead tasks result in major postural discomfort. These tasks are highly relevant in a variety of different branches [e.g., unskilled and skilled manual occupations (4)]. In this survey, it was revealed that 12.7% of participants suffer from non-ergonomic body postures from which 5.0% stated that overhead working tasks result in arm, neck, and shoulder pain. For overhead work, the risk of arm pain increases by 18% (4). It must be assumed that these and other manual tasks will remain essential in future work scenarios (5). At the same time, the number of qualified employees in many branches is decreasing (6). Therefore, it is essential to develop preventive programs or measures to reduce the number of WMSDs. Next to workplace health promotion including ergonomic or physical training, exoskeletons might be a potential solution to reduce WMSD.

Industrial exoskeletons designed for upper-body tasks, often referred to as upper-body exoskeletons or upper-limb exoskeletons, are wearable devices that assist and augment the movement and strength of the arms, shoulders, and torso (7). These exoskeletons can be used in various applications to enhance the performance of workers engaged in physically demanding jobs. They can be classified as assistive systems that provide mechanical support during activities such as lifting, reaching, and carrying objects. It has been shown that upper-body exoskeletons can reduce the physical strain on workers by providing support, promoting ergonomic posture and movement, and reducing the risk of fatigue (8–10).

However, wearing an exoskeleton can potentially impact cognition and motor control in several ways, although the effects can vary, based on the design of the exoskeleton, the individual using it, and the specific tasks being performed (11). Overall, the application of exoskeletons involves a psycho-physical interaction between the user and the support system. This interaction can be regarded as dual-task performance (DT), characterized as the performance of two simultaneous tasks integrating cognitive and motor resources (12). These interactions may lead to cognitive-motor interference (CMI) (12). CMI describes a performance decrement that occurs when two or more tasks (e.g., a working task and dealing with an exoskeleton) are executed simultaneously. Within studies that examined the cognitive load of exoskeleton use in industrial, military, or rehabilitation contexts CMI was observed for pace and reaction times (13). Moreover, the adaption to assistive devices can induce a higher attentional load (13, 14) and imposes greater effort in motor adaptation as well as neurocognitive control (15).

Recent reviews (7, 16, 17) examining the impact of upper-limb exoskeletons on cognitive workload and physical performance found that exoskeletons can have both positive and negative effects on cognitive workload and physical performance, depending on the type of task being performed and the design of the exoskeleton. For example, wearing an exoskeleton might require individuals to focus on managing the device, adjusting their movements, and maintaining balance (13). This could divert attention from other tasks or stimuli in the environment, affecting multitasking abilities. Moreover, while exoskeletons are designed to assist with movement, the interaction between the exoskeleton and the wearer’s body could change the way motor planning and coordination are executed (18). Users might need to adjust their usual movement patterns, potentially affecting the cognitive aspects of motor control.

Individual differences also play a role in how exoskeletons affect cognition and motor control. Some individuals may adapt quickly to wearing an exoskeleton, while others might experience more challenges. When individuals wear exoskeletons, there can be notable changes in EMG patterns due to the interaction between the exoskeleton and the wearer’s muscles. For example, exoskeletons are designed to provide mechanical assistance to muscles during movements. As a result, muscles might require less activation to perform tasks that are supported by the exoskeleton (19). This can lead to decreased EMG amplitudes in muscles directly affected by the assistance. Moreover, the exoskeleton might influence the coordination of muscle synergies, potentially altering the timing and activation levels of different muscles (11).

However, the impact of exoskeletons on EMG patterns can be task-specific (11). Different tasks might require varying levels of assistance and coordination, leading to different changes in muscle activation. This might also be influenced by fatigue. Exoskeletons can reduce muscle fatigue by assisting with tasks that would otherwise require high levels of effort (20). Exoskeletons are designed to provide mechanical support and assist wearers in performing physically demanding tasks, such as lifting heavy objects or maintaining strenuous postures. By sharing the load with the wearer’s muscles, exoskeletons can reduce muscle fatigue and strain. This could lead to reduced EMG fatigue-related changes during prolonged activities (19).

Monitoring EMG patterns while wearing exoskeletons provides valuable insights into how individuals interact with the device and adapt their movements. Understanding these changes is important for optimizing exoskeleton design, improving user comfort, and enhancing the overall effectiveness of exoskeleton technology for various applications.

Next, to the support of heavy loads, fine motor control inside the shoulder girdle plays a crucial role in many working conditions that require work at over-shoulder height. The shoulder joint is the most mobile joint in the human body, mostly held in place by tendons and the glenohumeral capsule. The surrounding muscles therefore act as both movers and stabilizers. Hence, to maintain joint stability, a well-balanced intermuscular coordination, involving not only the glenohumeral joint itself but also the scapula and other parts of the shoulder girdle is required (21). Prolonged work above shoulder level is a main contributor for the development of pathomechanisms that lead to shoulder pain or ailments such as impingement syndrome among others (22).

While movement speed and accuracy are two types of performances that can be improved or compromised declined by specific interventions like wearing an exoskeleton, managing both at the same time does represent a performance itself. Indeed, in work-related tasks, accuracy of movements is often constrained by time to improve efficacy and productivity. Moreover, it is a fact that performing a task that requires the fastest possible accuracy leads to a human making a trade-off that may result in lower speed when aiming for higher accuracy, and vice versa (23). This trade-off between speed and accuracy has been modelized for goal-directed arm movements by P.M. Fitts as a law which expresses that the duration of movement increases linearly with its complexity, i.e., its accuracy requirement (24, 25). Therefore, the more complex the task is, i.e., implies a high degree of accuracy, the more we need to slow the movement duration. The speed-accuracy trade-off of arm movements has been shown to be altered after an isometric muscle fatiguing task (26), as well as after a mentally fatiguing task (27).

To evaluate fine motor control, the tasks used in the Fitts’ law paradigm (24, 25) could add additional value when used to be integrated into studies on cognitive-motor interactions with exoskeleton use. Particularly, speed-accuracy trade-offs are highly relevant in several working conditions (eg. welding and assembly) but have been rarely examined with exoskeleton use (28).

Overall, the literature shows that upper-body exoskeletons are a promising way to reduce WMSDs. However, there are various aspects of motor control when using these exoskeletons that require further investigation. These include understanding potential variations in muscle activation patterns, changes in fine motor performance, and responses to additional tasks (DT). Furthermore, insights into these aspects, particularly in relation to fatigue and different work scenarios, are essential for determining the assistive capabilities of passive exoskeletons. In addition, it is still unclear how cognitive-motor resources are affected in the presence of an exoskeleton, during fatigue, and during different tasks, highlighting the need for further research in this area.

The interaction and their examination are rather complex and have not been executed so far. Therefore, this exploratory study aims to address the following research questions:

1. Which changes in muscle activity can be observed with exoskeleton use in a cognitive-motor DT situation before and after a fatigue protocol?
2. Which changes in fine motor control (speed-accuracy trade-off management) can be observed with exoskeleton use in a cognitive-motor DT situation before and after a fatigue protocol?
3. Does the cognitive performance in a cognitive-motor situation (nailing in over shoulder height with an auditory-verbal counting task) change with the use of a passive exoskeleton to provide shoulder support?

We hypothesized that using an upper-limb exoskeleton will (1) reduce muscle activity at the shoulder girdle in both the unfatigued and fatigued states. Based on this outcome, we expected (2) the motor performance to be increased by using the exoskeleton in a fatigued state. Regarding cognitive performance, we assumed that (3) exoskeleton usage would act as an additional attentional load, thereby decreasing the cognitive performance level.

## 2 Materials and methods

The pilot feasibility study took place at the facilities of the Department of Human Movement Science at the University of Hamburg in the summer of 2022. Ethical approval was obtained from the local ethics committee of the University of Hamburg (2021_417). Prior to the study, all participants signed written informed consent. The study followed the Declaration of Helsinki (version of 2013) and was registered at the German Clinical Trials Register (DRKS00032627).

### 2.1 Trial design

**Figure 1:**
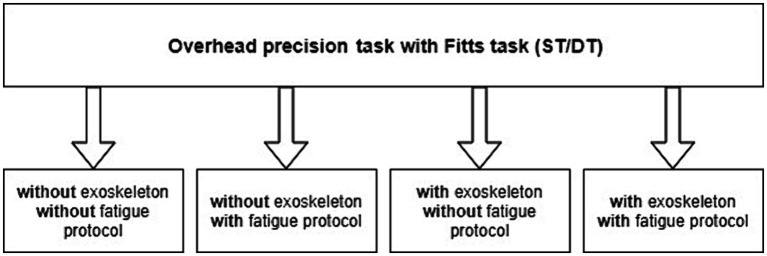
Study conditions (p. 5).

The study design was a randomized crossover study with repeated measurements (work activities with and without wearing the exoskeleton, before and after fatigue, under single- and dual-task conditions) under laboratory conditions (*cf.* Figure 1).

### 2.2 Participants

A total of 10 healthy participants (25.3 ± 2.5 years; 174.6 ± 10.2 cm; five female) were recruited from members of the University of Hamburg (employees and students) (*cf.* Table 1).

**Table 1:** Participants overview (p. 5).

	Age (years)	Height (cm)
Female (*n* = 5)	26 ± 3 (*n* = 3)	165 ± 7 (*n* = 5)
Male (*n* = 5)	25 ± 1 (*n* = 4)	183 ± 5 (*n* = 5)
Total (*N* = 10)	25 ± 3 (*n* = 7)	175 ± 11 (*n* = 10)

### 2.3 Experimental procedure

**Figure 2:**
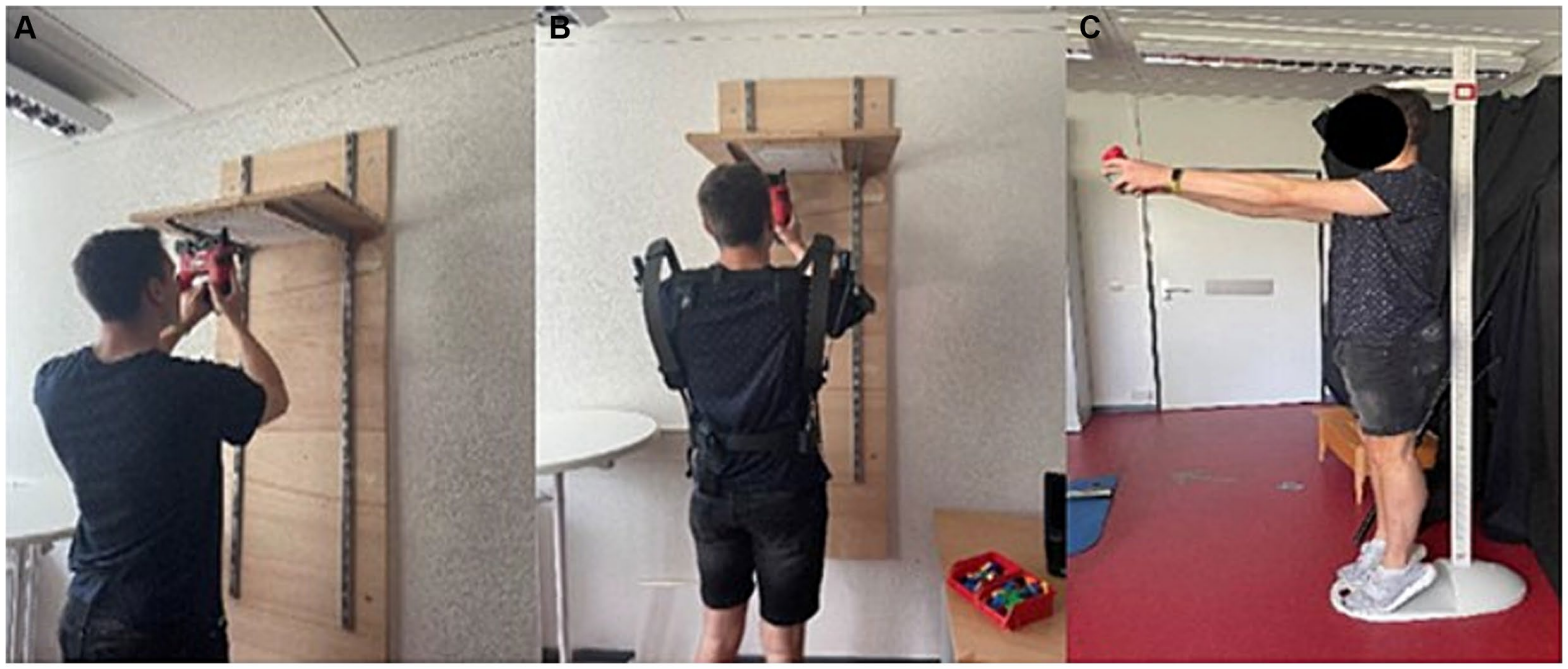
**(A)** Utilization of the nail gun without the exoskeleton. **(B)** Utilization of the nail gun with the exoskeleton. **(C)** Fatigue protocol (p. 5).

Overall, the participant’s task (single task [ST]) was to apply nails overhead with a nail gun in three predetermined difficulty levels based on Fitts’ law (further referred to as Fitts’ tasks) as quickly and accurately as possible. Depending on the randomization plan for the order of conditions, they performed the nailing task with and without an upper-body exoskeleton, as well as with and without previous physical fatigue based on the fatigue protocol. Within each of the four conditions, the nailing task was conducted with and without the additional secondary task in a randomized order. The secondary task was to audibly count all the beats of a metronome set at a frequency varying between 50 and 70 bpm throughout the entire task. Overall, the precision errors during nailing and the errors in counting the sound signals were recorded under both conditions (*cf.* Figure 2).

### 2.4 Overhead nailing under single-task (ST) and dual-task (DT) conditions using the Fitts’ task

The Fitts’ task is a standard motor control experiment in which participants move quickly between two targets of different sizes and distances (29). The aim is to assess the speed and accuracy of the target movements. The difficulty of the task is determined by a difficulty index, which is calculated based on the size and distance of the target areas. In this study, each stencil contained four targets (squares) at each corner. The size of each square and distance between squares have been set according to Fitts’ law equation to determine three indexes of difficulty: ID = log2 (2D/W) (ID: index of difficulty, D: distance between targets, W: width of each target). The three IDs have been set to increase linearly in difficulty (ID1 = 2.3; ID2 = 3.9; ID3 = 5.4).

The participants fulfilled the task in a standing position under a wooden board, equipped with the paper-based predetermined Fitts’ task patterns, into which they were supposed to place the nails. The height of the board was determined based on the condition that the elbows reached shoulder height as soon as the nail gun was in contact with the wooden surface. At a signal from the instructor, the participants began the task while the time measurement began (*cf.* Figure 2).

In the DT condition participants were asked to count the number of tones provided by a metronome with varying frequency around 50–70 bpm.

### 2.5 Exoskeleton usage

Participants wore a passive upper-body exoskeleton during the task, which uses the tension of elastic elements when the arm is lowered to provide the necessary energy for mechanical support when the arm is raised. The exoskeleton, which was used in this study (Skelex 360), was put on by a person according to the instructions, like a backpack. It was then adjusted based on anthropometric measurements indicated by numerical values. The aim is to position the support component on the upper arm as close as possible to the elbow without exceeding it. A uniform support force was selected for all participants and tasks. No further individual settings could be carried out by the participants (*cf.* Figure 2).

### 2.6 Fatigue protocol

Following previous research (11, 12), we decided to assess muscle activity of the shoulder as well as from the calf muscles by performing weight exercises in three sets. Each set consisted of a dynamic 2-min calf raise and a dynamic 2-min front raise exercise with weights. The men used a weight of 5 kg, the women a weight of 2.5 kg. A rest period of 60 s was observed between the individual sets. During the execution of the exercises, a metronome was set to a tempo of 60 beats per minute (bpm), and both the front lift and the calf raise were to be performed at this tempo (*cf.* Figure 2).

### 2.7 Measurements/test instruments

The primary outcomes of the study were muscle activity and the time taken to place a nail into a square in each Fitts’ task, as well as the error rates in the DT scenario for the auditory counting task. The main focus was on the difference induced by the use of the upper-body exoskeleton as well as by previous fatigue.

#### 2.7.1 sEMG

For this study, the muscle activity of the back muscles (*M. erector Spinae* thoracic region) and the upper back and dominant arm muscles (*M. deltoideus* pars clavicularis, *M. trapezius* pars descendens) were recorded. The selected muscles play an essential role in spinal stabilization, shoulder girdle movement, and arm elevation during overhead work. This activity was monitored to assess the effects of using an upper-body exoskeleton on muscle activation patterns and fatigue in these critical anatomical regions. To achieve this, the participants were fitted with appropriate electrodes (myon aktos, myon AG, Switzerland) before the tasks were carried out and the maximum voluntary contraction (MVC) tests for the respective muscles were performed according to the SENIAM guidelines (30), and the instructions by Konrad (31).

First, the sEMG data were screened before the amplitude normalization of the previously collected MVC data. Afterward, the normalized data were filtered with a Butterworth high and low pass filter (20–400 Hz), rectified and the RMS was calculated so that the data of the participants could be compared with each other in terms of percentage.

### 2.8 Statistical analysis

The following statistical analysis were conducted in a fully blinded procedure with references to the research questions.

However, it should be noted that possible confounding factors, such as previous experience with exoskeletons or individual physical condition, were not included in the statistical analysis. However, this was asked in advance and all participants denied any previous experience with exoskeletons. Physical condition was also asked for, and the majority of participants were sports students, so physical condition tended to be high.

#### 2.8.1 sEMG

Since a normal distribution of the data could be assumed via the Kolmogorov–Smirnoff test, repeated measures ANOVA was conducted comparing (1) ST and DT as well as (2) with and without upper-body exoskeleton and (3) with and without fatigue protocol following the recommendations by Blanca et al. (32). Furthermore, we conducted Bonferroni *post-hoc* tests for between-subject differences for scenarios 1–3. All data were processed with SPSS 29.0 and analyzed at the significance level *α* = 0.05.

#### 2.8.2 Speed-accuracy trade-off (duration of Fitts’ task)

**Figure 3:**
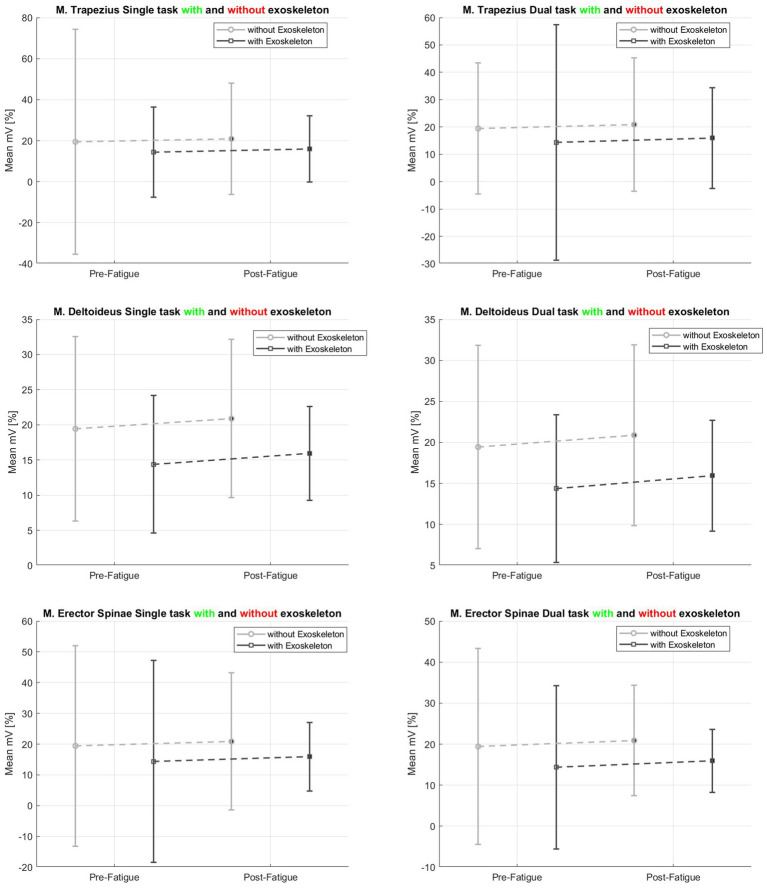
Fitts’ law relationships in the different conditions, pre- and post-fatigue (p. 8).

Time to perform each Fitts’ task has been measured. Then, each time performance has been plotted against its corresponding index of difficulty (*cf.* Figure 3) to build the Fitts’ law regression line. A four-way repeated measure ANOVA was performed on times to perform Fitts’ tasks, with factors *type of task* (single, dual) *exoskeleton* (with, without), *fatigue* (pre- and post-fatiguing exercise), and *index of difficulty* (ID1, ID2, and ID3). Moreover, the number of mistakes for each condition was analyzed. In case of a significant effect, a *post-hoc* test with Bonferroni correction was performed. In addition, effect sizes were quantified. Partial-eta-squared (η*_p_*^2^) was calculated from ANOVA results, with values of 0.01, 0.06, and above 0.14 representing small, medium, and large differences, respectively.

Finally, the slope of the relationship between movement time and index of difficulty has been calculated for each condition. A three-way repeated measure ANOVA with factors *type of task* (single, dual) *exoskeleton* (with, without), *fatigue* (pre- and post-fatiguing exercise) has been performed on slopes.

## 3 Results

The results of the study are described in the following, separated by muscle activity, speed-accuracy trade-off management (Fitts’ task) with and without exoskeleton according to the fatigue state and cognitive performance within the test conditions.

### 3.1 Muscle activity in all conditions (sEMG)

**Figure 4:**
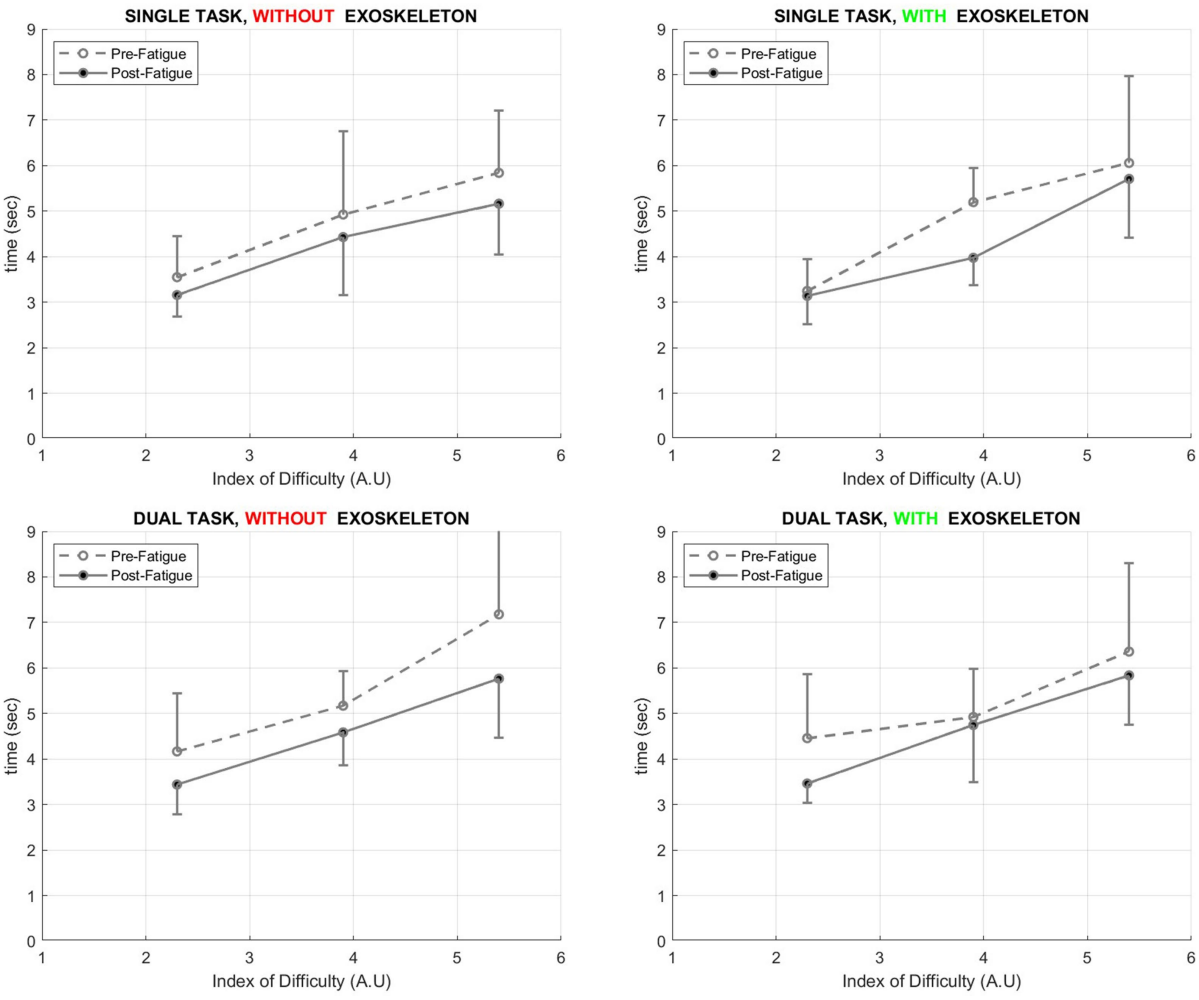
sEMG data (p. 8).

For the *M. deltoideus* as well as for the *M. trapezius* significant reductions from ST to DT were observed (*M. deltoideus*: *F* (1, 8) = 8.672, *p* = 0.019, η*_p_*^2^ = 0.520; *M. trapezius*: ST–DT *F* (1, 8) = 17.268, *p* = 0.003, η*_p_*^2^ = 0.683). Moreover, for the exoskeleton use, the reduction of the muscle activity of the *M. deltoideus* failed to be significant (*F* (1, 8) = 4.849, *p* = 0.059, η*_p_*^2^ = 0.377) (*cf.* Figure 4). There were no differences between all examined conditions for the *M. erector spinae* (*cf.* Table 2 and Figure 4).

**Table 2:** Muscle activity (sEMG).

			ST (%)	DT (%)	Main effects	Interaction effects
D	Exo	No fatigue	14.9 ± 10.2	14.0 ± 9.5	ST–DT *F* (1, 8) = 8.672, *p* = 0.019, η*_p_*^2^ *=* 0.520 Exo *F* (1, 8) = 4.849, *p* = 0.059, η*_p_*^2^ *= 0.377* Fatigue *F* (1, 8) = 0.398, *p* = 0.546, η*_p_*^2^ = 0.047	Task × Exo *F* (1, 8) = 0.003, *p* = 0.958, η*_p_*^2^ < 0.001 Task × Fatigue *F* (1, 8) = 1.296, *p* = 0.288, η*_p_*^2^ = 0.139 Exo × Fatigue *F* (1, 8) = 0.109, *p* = 0.750, η*_p_*^2^ = 0.013 Task × Exo × Fatigue *F* (1, 8) = 0.573, *p* = 0.470, η*_p_*^2^ = 0.067
		Fatigue	16.2 ± 7.0	14.7 ± 7.1		
	No exo	No fatigue	18.8 ± 13.8	18.5 ± 13.1		
		Fatigue	21.4 ± 11.8	19.2 ± 11.4		
T	Exo	No fatigue	23.4 ± 22.8	22.5 ± 25.6	ST–DT *F* (1, 8) = 17.268, *p* = 0.003, η*_p_*^2^ = 0.683 Exo *F* (1, 8) = 1.537, *p* = 0.250, η*_p_*^2^ = 0.161 Fatigue *F* (1, 8) = 1.226, *p* = 0.300, η*_p_*^2^ = 0.133	Task × Exo *F* (1, 8) = 3.050, *p* = 0.119, η*_p_*^2^ = 0.276 Task × Fatigue *F* (1, 8) = 0.957, *p* = 0.357, η*_p_*^2^ = 0.107 Exo × Fatigue *F* (1, 8) = 0.961, *p* = 0.356, η*_p_*^2^ = 0.107 Task × Exo × Fatigue *F* (1, 8) = 0.853, *p* = 0.383, η*_p_*^2^ = 0.096
		Fatigue	22.1 ± 17.1	22.1 ± 19.2		
	No exo	No fatigue	47.5 ± 57.4	23.7 ± 25.3		
		Fatigue	26.4 ± 28.4	36.3 ± 45.7		
ES	Exo	No fatigue	20.6 ± 34.7	15.2 ± 21.0	ST–DT *F* (1, 8) = 1.574, *p* = 0.245, η*_p_*^2^ = 0.164 Exo *F* (1, 8) = 0.666, *p* = 0.438, η*_p_*^2^ = 0.077 Fatigue *F* (1, 8) = 1.168, *p* = 0.311, η*_p_*^2^ = 0.127	Task × Exo *F* (1, 8) = 0.528, *p* = 0.488, η*_p_*^2^ = 0.062 Task × Fatigue *F* (1, 8) = 0.969, *p* = 0.354, η*_p_*^2^ = 0.108 Exo × Fatigue *F* (1, 8) = 0.411, *p* = 0.539, η*_p_*^2^ = 0.049 Task × Exo × Fatigue *F* (1, 8) = 1.315, *p* = 0.285, η*_p_*^2^ = 0.141
		Fatigue	12.8 ± 10.6	11.0 ± 7.3		
	No exo	No fatigue	19.2 ± 30.9	15.6 ± 22.7		
		Fatigue	16.6 ± 21.1	12.1 ± 12.8		

D = M. deltoideus, T = M. trapezius, ES = *M. erector spinae*, ST = single task, DT = dual task, task = ST and DT, exo = exoskeleton.

In addition to Table 2, Figure 4 shows the mean and standard deviation for each muscle in the percentage of MVC between pre- and post-fatigue and for single and dual tasks separately.

If also controlling for gender, *post-hoc* analysis showed significant differences for *M. deltoideus* across all conditions (*F* (1, 8) = 7.618, *p* = 0.025, η*_p_*^2^ = 0.488). Looking at the individual differences for the conditions, the results showed the following significant differences in the one-way ANOVA for the *M. deltoideus*: single-task post-fatigue without exoskeleton (*F* (1, 9) = 8.154, *p* = 0.021, η*_p_*^2^ = 0.505), single-task pre-fatigue with exoskeleton (*F* (1, 9) = 6.113, *p* = 0.039, η*_p_*^2^ = 0.433), dual-task pre-fatigue without exoskeleton (*F* (1, 9) = 6.295, *p* = 0.036, η*_p_*^2^ = 0.440), and dual-task post-fatigue without exoskeleton (*F* (1, 9) = 8.387, *p* = 0.020, η*_p_*^2^ = 0.512). For the conditions of single-task pre-fatigue without exoskeleton and dual-task pre-fatigue with exoskeleton, the results showed barely insignificant values.

### 3.2 Speed-accuracy trade-off management with and without exoskeleton according to the fatigue condition (Fitts’ task)

Figure 3 shows the differences between the fatigue and the IDs (Fitts’ task) separately for the conditions with and without the exoskeleton. For the ST condition, no main effect of the *exoskeleton* was found (*F* (1, 8) = 0.112, *p* = 0.745, η*_p_*^2^ = 0.012), independent of the index of difficulty (ID) or the level of fatigue. A main effect of *fatigue* was found during ST conditions (*F* (1, 8) = 6.378, *p* = 0.032, η*_p_*^2^ = 0.415), regardless of ID or exoskeleton use (*cf.* Figure 4). In addition, a main effect was found for ST for the *ID* (*F* (1, 8) = 58.89, *p* < 0.001, η*_p_*^2^ = 0.867), with each ID leading to a significantly longer movement time than the lower one.

In the dual-task scenario, similar to single task, no main effect of *exoskeleton* was found (*F* (1, 8) = 0.837, *p* = 0.386, η*_p_*^2^ = 0.094), but a main effect of *fatigue* (*F* (1, 8) = 5.880, *p* = 0.041, η*_p_*^2^ = 0.423) and *ID* (*F* (1, 8) = 30.116, *p* < 0.001, η*_p_*^2^ = 0.79). A main effect of *task* was found for the times to complete the Fitts’ tasks (*F* (1, 8) = 13.415, *p* = 0.005, η*_p_*^2^ = 0.598). The time was always higher in the dual-task conditions. Finally, neither a main effect nor an interaction (*F* (1, 8) = 1.793, *p* = 0.213, η*_p_*^2^ = 0.166) was found for the slopes of Fitts’ law calculated for each condition (pre and post-fatigue, with or without the use of an exoskeleton, during the single or dual task).

### 3.3 Cognitive performance (errors) within the experimental conditions

The number of errors between the four conditions (1) working without exoskeleton (mean errors 2.2 ± 1.4), (2) working with exoskeleton (mean errors 2.5 ± 2.3), (3) working without exoskeleton after fatigue (mean errors 2.3 ± 2.4) and (4) working with exoskeleton after fatigue (mean errors 0.8 ± 0.9) showed a mean difference (*F* (3, 7) = 4.618, *p* = 0.044, η*_p_*^2^ = 0.664). *Post-hoc* analysis showed that the number of errors in condition four was significantly lower than in all other conditions.

## 4 Discussion

The purpose of this exploratory pilot study was to examine the influence of exoskeleton use and fatigue on cognitive-motor resources during an overhead nailing task. Specifically, we wanted to examine changes in muscle activity, motor, and cognitive performance with or without exoskeleton use in a cognitive-motor DT situation before and after a fatigue protocol. We hypothesized that using an exoskeleton will reduce muscle activity and increase motor performance after fatigue but decrease cognitive performance. Moreover, this study first integrated the concept of speed-accuracy trade-off management in combination with dual tasking into the experimental design.

### 4.1 Muscle activity

The results of the muscular activity showed diverging results, which are, however, consistent with the results of previous studies by Gillette et al. (33), which found a significant reduction in muscle activity of the *M. deltoideus* by using an upper-body exoskeleton, as well as with the review by Bär et al. (11). A 35% reduction in the activity of the *M. deltoideus* was observed, without considering the task or previous fatigue caused using the exoskeleton. In addition, a reduction of up to 52% in the activation of the *M. trapezius* and up to 23% in the activation of the *M. erector spinae* was also observed when using the exoskeleton, however, these observations failed to be significant. Therefore, we conclude that according to the results of the review by Bär et al. (11) that muscle activation might be task-specific and is also related to the weight of the nail gun tool.

Another potential reason might be the small sample size with an equal number of male and female participants. Within the study design of Kermavar et al. (34) especially the female participants had greater reductions in shoulder muscle activity. Regarding all task conditions, female participants showed an average of 12% higher muscle activation of the *M. deltoideus*. This could be due to the anthropometric differences between men and women, as exoskeletons today are still strongly adapted and designed for male anthropometry. Given the fact, that women have so far received little to no attention in research on supporting upper-body exoskeletons, this is an important step toward further understanding of the interaction between the body from an anthropometric view and an exoskeleton, which ideally adapts to the different anthropometric conditions and the needs of the user. Furthermore, it is necessary to understand these interactions to find out which measures need to be taken to optimally adjust the support of the female body, so that women can also benefit from exoskeletons.

A third explanation addresses the level of support. The review by Theurel and Desbrosses (20) showed that the use of a passive exoskeleton might induce unexpected consequences on the coordination between agonist and antagonist muscles. According to the findings by Kermavnar et al. (34), we were not able to determine a reduction in the activation of the *M. erector spinae*, but Walter et al. (35) found comparable results regarding the lack of significance between working with and without a passive exoskeleton with significant benefits of an active exoskeleton. This supports the idea that the level of support plays a decisive role, which needs to be examined in more detail in future studies in order to evaluate an optimal level of support (7).

Considering differences in muscular activity between single- and dual-task conditions, this study showed a significant reduction in the shoulder muscles of the *M. deltoideus* in the dual-task condition of 7–10% compared to the single-task condition, which could be an indication of lower motor control depending on the change in attentional resources (e.g., according to Wickens et al. (36)). Furthermore, reductions in DT conditions of up to 54% for the *M. trapezius* and up to 43% reduction in the *M. erector spinae* were observed using an exoskeleton even if there were no significant differences between the conditions (with and without exoskeleton) (*cf.* Table 2).

If we look at the differences between the use of an exoskeleton within the pre- and post-fatigue conditions, our study shows a reduction in muscular activation of the *M. deltoideus* in ST conditions of up to 26% without prior fatigue. With fatigue, there was an even greater effect of 32% reduction. Furthermore, a reduction of up to 53% in the muscular activation of the *M. trapezius* was shown when using the exoskeleton even without prior fatigue; with fatigue, the reduction was only 40%. For the *M. erector spinae*, the use of the exoskeleton also showed an effect without fatigue, with a reduction in muscular activation of up to 21% and with fatigue of up to 34%, which are similar to findings by Wei et al. (37) (*cf.* Table 2). This once again highlights the positively supportive effect of the upper-body exoskeleton on reduced muscular activation, particularly the enhanced supportive effect following a period of physical exertion, such as encountered in a workday characterized by overhead tasks in the industrial setting. This decrease in muscular activation signifies a diminished demand on the musculoskeletal system, especially the upper extremity and the shoulder girdle (21, 22). Consequently, using upper-body exoskeletons serves as a preventive factor for the reduction of work-related musculoskeletal disorders, especially in overhead tasks.

In summary, it is most reasonable to assume that the included participant sample size was too small to be able to determine a significant difference in the *M. erector spinae* for example. On the other hand, familiarization with the exoskeleton could also have played a decisive role, which, according to Walter et al. (35), is crucial for the positive impact of the exoskeleton. Our study population did not have any experience with upper-body exoskeletons prior to the beginning of this study and only underwent a brief familiarization phase at the start of the study. Nevertheless, this aspect reflects real-world scenarios, when workers will start using exoskeletons, as to this date most employees have no prior exoskeleton experience. This emphasizes the need for high-quality studies with trained and experienced exoskeleton users to demonstrate a significant reduction in muscular activity due to the potential benefits of the exoskeleton.

### 4.2 Speed-accuracy trade-offs

As predicted by the law of Fitts, drilling movement duration was linearly increased as the index of difficulty increased (24, 25). Indeed, the nature of the relationship between movement duration and task complexity is usually mathematically expressed as a linear relationship. However, this has been shown for table and pen tasks, such as linear pointing movements (25) or circular drawing tasks (29). In general, it has been known for more than two decades that a wide variety of rapid aimed movements does respect Fitts’ law (38). However, the use of ecological tasks is very rare in the literature, and Fitts’ law has never been evaluated in drilling tasks such as in the present study. Therefore, it is interesting to note that Fitts’ tasks usually performed in a laboratory setting on a paper-to-pen test can be transferred to an ecological upper-body task. This opens a wide new range of research in the field of ergonomics and the study of motor control of work-related tasks. This is utterly important since daily activities involve fine motor skills to a greater extent than standardized laboratory tasks and involve a higher level of cognitive process (39).

Interestingly, the present study also showed the feasibility of using a Fitts’ task while wearing an exoskeleton, since the Fitts’ law was still respected. The observation is even more striking, since the use of an exoskeleton did not affect the Fitts’ relationships, whatever the level of fatigue or cognitive requirement. This was expressed by identical movement durations in all indexes of difficulties and slopes of Fitts’ relationships. In patients, it is known that wearing an exoskeleton may alter movement accuracy, especially with the heaviest exoskeletons (40). Conserving a smooth movement using an exoskeleton, whether it is a passive or active model, often represents a huge challenge for companies in all fields, including industrial and medical (41). The present study raised that (1) healthy young participants can easily adapt to the use of an exoskeleton to perform a fast and accurate task, and (2) wearing an exoskeleton (at least for the model used in the present study), does not alter the ability to manage the speed-accuracy trade-off.

In the present study, Fitts’ law was also not affected by muscle fatigue in its form, but a global decrease in MT has been observed. It has been shown that muscle fatigue was more prone to induce a global increase in movement time during a classical Fitts’ pointing task (26). However, in the study of Missenard et al., a decrease in muscle force of 30% was necessary to induce a change in movement duration. Therefore, it is possible that the present study did not induce sufficient muscle fatigue to decrease muscle performance. Moreover, it is known that there is a specificity of the fatiguing exercise to the following changes observed in the neuromuscular system (42). In other terms, the lack of direct correspondence of the fatiguing exercise and the post-task (Fitts’ drilling task), despite a correspondence in solicited muscle groups, may lead to a lack of muscle fatigue in the present study, or even the opposite, an increase in performance (e.g., a decrease in movement time in Fitts’ task). Then, this latter may be due to an overcompensation or a change in muscle activation strategies. It is well known that the motor system can adapt its strategy to compensate for muscle fatigue, to preserve the Fitts’ law behavior, as observed by a similar slope before and after fatigue (26). In other words, after a fatiguing exercise, the Fitts’ law could be altered only quantitatively, but not qualitatively. Interestingly, Missenard et al. did not find any changes in EMG activity of agonist or antagonist muscles between pre- and post-fatigue, despite a global increase in movement duration during the Fitts task. Again, this shows the ability of the system to adapt to any fatiguing exercise, and that neither EMG nor movement parameters could solely be considered to account for neuromuscular fatigue.

Finally, a globally greater movement duration was observed in dual task as compared to single task, as evidenced by an upward shift of Fitts’ law in all conditions (with/without exoskeleton, with/without fatigue). This shows that the lack of cognitive resources may lead to an increase in movement time, whatever the index of difficulty of the task. Resource allocation to the DT may lead the motor system to make the choice to slow down the movement to be able to process the cognitive task properly. It has been shown for instance that in the presence of mental fatigue, a state that also generates a decrease in cognitive resources, an upward shift in Fitts’ law (e.g., a global increase in movement duration) was also observed (27). According to motor control theories, the central nervous system, mainly at the stage of motor planning, may adopt this strategy of slowing down the movement to preserve task success (43). However, it should be noted that, despite this upward shift, Fitts’ slope relationship was not altered by the dual task. Then, after muscle fatigue, the shape of the Fitts’ law was altered quantitatively but not qualitatively. Therefore, this shows again that the system could adjust movement duration similarly for each index of difficulty to preserve its ability to manage the speed-accuracy trade-off. For generalization, these results should be confirmed with larger sample sizes.

### 4.3 Cognitive performance

In contrast to the results of Govaerts et al. (44), Bequette et al. (13), Federici et al. (14), and Zhu et al. (15), our results of cognitive performance, measured by the error frequencies in the different conditions, clearly show a clear advantage in working with an exoskeleton, especially after physical fatigue, which should be comparable to a working day following the fatigue protocol by Bär et al. (11), and Leone et al. (12). A 65% reduction in errors was recorded compared to working without an exoskeleton (see the section “Cognitive performance (errors) within the test conditions”). This could be a compensatory effect of the exoskeleton, or the complaints were not perceived in the fatigue state. One might now also assume that learning effects could possibly be responsible for this reduction, but this can be rejected as the trials were carried out in randomized order. In addition, no task prioritization effect could be observed based on the results of the Fitts’ task.

In summary understand all changes in cognitive-motor control and resource allocation discussed with respect to our study findings we recommend using study designs, such as those by Zhu et al. (15), to examine brain activity (e.g., fNIRS, EEG) to gain more insight into the shift in the attentional focus.

In addition, further studies based on the muscle synergy approach are much needed, as this method considers how different muscle groups interact to produce coordinated movements. Following this framework will allow a better understanding of the impact of wearing an exoskeleton in terms of movement coordination, the control of specific muscle groups, and the cognitive processes involved in managing these movements. Since these factors have a significant influence on the effectiveness of exoskeleton use, further research will provide valuable insights for the optimization of exoskeleton designs and their use in different applications. Finally, it would be beneficial to also monitor heart rate or heart rate variability within the experimental design to get insights into physical exhaustion that might impact the different performance levels.

The findings of this study furthermore underline the importance of a holistic view of exoskeleton usage in the field in which not only its potential physiological benefits during motion activities are to be considered, but also how this human–machine coupling will affect other areas such as attentional resources and thereby productivity in the long run as well. Exoskeleton integration into the workplace should therefore not only aim for a general familiarization of its usage but furthermore identify areas in which exoskeletons can have the potential of lowering attentional stress (or place an additional burden for that matter) in order to maintain productivity in conjunction with workplace safety.

## 5 Limitations

The present study results are constrained by a few limitations that should be noted when generalizing effects. First, the included study participants led to a comparatively small sample size as well as the homogeneity of the participants in terms of physical fitness and their work as students, which may lead to these concerns regarding the generalizability of the results. Indeed, this limits the external validity of the present results to industrial workers, which represent a more heterogenous population, in terms of age or physical condition. Future studies should transfer the study design into more ecologically valid real-world scenarios. The statistical power should be considered carefully by sample size calculation *a priori*.

Secondly, the lack of measuring individual perceived exertion via RPE limits our understanding of participants’ subjective experiences during executing the tasks, especially the experiences during using the exoskeleton. Future studies should pay more attention to this aspect as it is a key indicator that provides insights into the effectiveness and usability of the intervention as well as on the usage of the exoskeleton. It may also be possible to monitor the heart rate using wearable monitoring systems (e.g., on the wrist or with a chest strap).

Furthermore, only physical fatigue was recorded in this study using the sEMG of selected muscles. Future studies should also evaluate cognitive fatigue to obtain a broader statement on general stress and should also focus on the long-term exposure of users.

Finally, the use of a passive exoskeleton in our study means that participants were unable to adjust and actively control the exoskeleton support. This stands in contrast to studies using exoskeletons with active support functionalities where users can adapt the support to the requirements of the task as well as their individual needs. Consequently, the results of our study could be influenced by the lack of user-driven support.

Following this, no assessments of postural discomfort were made in this study, leading to the fact that it limits our ability to fully evaluate the ergonomic effects of the exoskeleton. Understanding participants’ comfort/discomfort is essentially for optimizing the exoskeleton designs as well as integrating different functionalities and improving adaptation for example to the needs of female users.

## 6 Conclusion

Despite the potential limitations and the lack of generalizability, this study can contribute to a better understanding of muscular, cognitive, and motor demands as well as speed-accuracy trade-off management and cognitive performance enhancement. In addition, the indications of differences between female and male exoskeleton users in muscular demands may represent important focuses for future research and generalizability of the effects. Furthermore, user-specific interactions with the exoskeleton are discussed with respect to generalizability.

## Data availability statement

The raw data supporting the conclusions of this article will be made available by the authors, without undue reservation.

## Ethics statement

The studies involving humans were approved by the Local Ethics Committee of the Faculty PB of the University of Hamburg. The studies were conducted in accordance with the local legislation and institutional requirements. The participants provided their written informed consent to participate in this study. Written informed consent was obtained from the individual(s) for the publication of any potentially identifiable images or data included in this article.

## Author contributions

JG: Conceptualization, Data curation, Formal analysis, Methodology, Visualization, Writing – original draft. SG: Data curation, Formal analysis, Methodology, Writing – review & editing. AA-W: Data curation, Formal analysis, Methodology, Writing – review & editing. BW: Conceptualization, Data curation, Formal analysis, Methodology, Supervision, Writing – original draft.
